# Pseudovirus-mediated proximity labeling identifies candidate host cell membrane proteins involved in viral attachment

**DOI:** 10.1128/jvi.00507-26

**Published:** 2026-04-29

**Authors:** Norihiro Kotani, Kensuke Iwasa, Tomoko Amimoto, Chikara Yamashita, Kumiko Komatsu, Yutaka Narimichi, Yoshiki Wakabayashi, Yuuki Kurebayashi, Yutaka Horiuchi, Leona Kashimata, Ryoko Sasaki, Megumi Kumagai, Nan Yagishita-Kyo, Takeo Awaji, Takashi Murakami, Yosuke Mizuno, Miyako Nakano, Tadanobu Takahashi, Hideyuki Takeuchi, Koichi Honke

**Affiliations:** 1 Department of Pharmacology, Saitama Medical University 13031, Iruma-gun, Saitama, Japan; 2 Medical Research Center, Saitama Medical University 13031, Iruma-gun, Saitama, Japan; 3 Department of Biochemistry, Saitama Medical University 13031, Iruma-gun, Saitama, Japan; 4 Natural Science Center for Basic Research and Development, Hiroshima University 12803, Higashi-Hiroshima, Hiroshima, Japan; 5 Biomedical Research Center, Saitama Medical University 13031, Iruma-gun, Saitama, Japan; 6 Department of Biochemistry, School of Pharmaceutical Sciences, University of Shizuoka 73830, Shizuoka, Shizuoka, Japan; 7 Department of Microbiology, Saitama Medical University 13031, Iruma-gun, Saitama, Japan; 8 Graduate School of Integrated Sciences for Life, Hiroshima University 12803, Higashi-Hiroshima, Hiroshima, Japan; 9 Department of Biochemistry, Kochi University Medical School 528625, Nankoku, Kochi, Japan; St Jude Children's Research Hospital, Memphis, Tennessee, USA

**Keywords:** proximity labeling, membrane protein, virus attachment, pseudovirus, influenza virus, SARS-CoV-2

## Abstract

Viral infections pose a significant threat to humanity, as exemplified by the COVID-19 pandemic. To mitigate the associated damage, understanding the biological characteristics of causative viruses is essential. In this study, we report a novel method for identifying host cell molecules (including virus receptors) required for viral attachment, referred to as host cell attachment factors (HCAFs). Our approach utilizes proximity labeling (PL) technology; pseudoviruses engineered to express a PL enzyme are applied to host cells, where they bind and initiate PL. Because HCAFs are expected to localize near viral attachment sites, candidate HCAFs were specifically tagged using the PL process. The analysis of influenza HCAFs enabled the identification of multiple candidate molecules. Subsequent viral attachment experiments using Chinese hamster ovary cells suggested that neuropilin-1 may serve as an HCAF for influenza viruses. Given its simplicity and rapid turnaround, this method is adaptable to a wide range of enveloped viruses, including pandemic viruses that require emergency analyses.

**IMPORTANCE:**

Identifying cellular receptors for viruses is not only crucial in conventional virology research but also plays a key role in addressing pandemic viruses. The significance of our work lies in establishing a standardized method for viral receptor identification, an area in which no widely accepted protocol has previously existed, by utilizing pseudovirus-mediated proximity labeling technology. Moreover, this approach provides a highly versatile platform that enables rapid and low-cost identification of viral receptors.

## INTRODUCTION

The emergence of pandemic viruses poses a significant threat to humanity, as exemplified by the COVID-19 (SARS-CoV-2) pandemic, which began in 2020 and caused extensive global damage (1, 2). Such viral crises are expected to recur in the future. For example, the emergence of novel pandemic strains of influenza viruses (family Orthomyxoviridae) remains a major concern (2–4). To mitigate the impact of these crises, understanding the biological characteristics of causative viruses is essential. In particular, the identification and analysis of primary viral receptors and coreceptors involved in viral entry into host cells (5–8) provide crucial insights for the development of vaccines and antiviral agents.

In the case of SARS-CoV-2, angiotensin-converting enzyme 2 (ACE2) was rapidly identified as the primary receptor (7) based on previous studies of SARS-CoV-1, which caused an epidemic in the early 2000s (9). However, it is not guaranteed that future pandemics will follow similar patterns. Furthermore, SARS-CoV-2 has been reported to enter cells lacking ACE2 expression, and although several candidate coreceptors (cofactors) have been proposed (10, 11), their precise roles remain unclear. Therefore, rapid identification and characterization of host cell molecules necessary for viral attachment, referred to here as host cell attachment factors (HCAFs), which are not limited to SARS-CoV-2, will be increasingly important for preparedness against pathogenic and future pandemic viruses.

We previously developed a proximity labeling (PL) technique known as enzyme-mediated activation of radical sources (EMARS), which utilizes horseradish peroxidase (HRP)-mediated radical reactions to biochemically identify molecular complexes formed on biological membranes under physiological conditions (12, 13). This technique enables the specific labeling of molecules that are present as complexes in close proximity to a target cell membrane molecule to which HRP has been conjugated via an antibody or a similar approach. Following purification of the labeled molecules, proteomic analysis can be performed to identify the components of these molecular complexes. This is now recognized as a pioneering technology in the field of PL and has been applied to the study of molecular interactions and complexes (14–22).

Based on these advances, we hypothesized that the spike protein of a virus, upon attachment to its primary receptor and localization near HCAFs on the host cell membrane, forms molecular complexes that can be collectively labeled and identified using EMARS-based proteomic analysis. This approach has enabled the comprehensive identification of candidate HCAF for SARS-CoV-2. Using this approach, we demonstrated that several membrane proteins, including DPP4, cadherin-17, and CD133, form complexes with the SARS-CoV-2 spike protein (S1-RBD) on the surface of highly susceptible Caco-2 cells, suggesting their involvement in SARS-CoV-2 infection (23). Thus, EMARS represents a promising tool for identifying HCAF in various viruses, including pandemic viruses. This method allows for the rapid and straightforward listing of candidate molecules within a few weeks, provided that the spike protein (or its binding domain recombinant protein) of the target virus is available. This represents an unprecedented, rapid, and convenient approach for HCAF analysis. Moreover, because the method is not restricted to viral species if the spike protein is available, it is expected to be applicable to a wide range of existing viruses.

However, this approach relies on recombinant spike proteins. The synthesis and purification of recombinant proteins require a certain amount of time. In addition, performing EMARS under actual viral infection conditions would be more physiologically relevant and applicable to diverse viruses.

In this study, we established a system in which EMARS can be performed during the binding of non-recombinant spike proteins but PL virus to host cells. PL viruses were produced using (i) GPI anchor-fused HRP (14, 15)-expressing HEK293T packaging cells or (ii) simultaneous transfection with GPI-anchor-fused HRP and viral genes into typical HEK293T packaging cells. PL viruses expressing the spike proteins of VSV-G, SARS-CoV-2, and influenza viruses successfully bound to host cells and induced EMARS. Notably, in an analysis of the influenza virus, neuropilin-1 (NRP1) was identified as an HCAF. While sialic acid and surface glycans have long been proposed as primary receptors for influenza viruses (24, 25), our study suggests that NRP1 may serve as a novel HCAF involved in influenza virus attachment to host cells.

## RESULTS

### Production and characterization of packaging cells expressing HRP

To generate lentivirus-based HRP-expressing viruses for PL, we adopted a method in which HRP is first expressed directly in packaging cells, followed by the introduction of viral vectors (two-step method; Fig. S1A). Initially, we constructed an HRP-expressing lentiviral vector by cloning the ORF of human codon-optimized HRP with the signal sequence and the GPI-anchor region of complement decay-accelerating factor (P08174; DAF) or Thy1 (P04216; THY) into a pLenti-based plasmid. Using these vectors, we produced lentiviral particles for HRP expression. These viruses were then used to infect HEK293T cells and stable cell lines expressing HRP were established through antibiotic (puromycin) selection. The established HRP-stable HEK293T cells were cultured and HRP expression was confirmed (Fig. S1B through D). Fluorescent staining was observed in both DAF-HRP and THY-HRP packaging cells (Fig. S1B). However, higher HRP expression was detected in DAF-HRP packaging cells than in THY-HRP packaging cells (Fig. S1B and D). FACS analysis of DAF-HRP packaging cells revealed that at least approximately 30% of cells were highly expressed in population (P3 region; Fig. S1C). In addition, western blot analysis showed stronger HRP expression in DAF-HRP packaging cells (Fig. S1D; left area).

### Production and characterization of pseudoviruses generated by the two-step method

Next, the pseudoviruses produced from both HRP-expressing packaging cell lines were analyzed using western blotting to confirm HRP incorporation. HRP was detected in enriched pseudoviruses from both DAF-HRP and THY-HRP packaging cells, but pseudoviruses derived from DAF-HRP packaging cells showed a modestly higher HRP signal intensity than those from THY-HRP packaging cells (Fig. S1D; right area). To examine the infectivity of the pseudoviruses, VSV-G pseudoviruses for GFP expression were produced from DAF-HRP or THY-HRP packaging cells, enriched, and then applied to HEK293T cells that had been cultured separately as host cells. After 48 h, fluorescence microscopy revealed that VSV-G pseudoviruses produced using conventional HEK293T packaging cells infected the majority of the cells, resulting in GFP-positive cells (Fig. S2A; upper column). In contrast, GFP-expressing VSV-G pseudoviruses produced from DAF-HRP or THY-HRP packaging cells exhibited a reduced number of GFP-positive cells, indicating a decrease in infection efficiency (Fig. S2A; middle and lower columns).

The EMARS reactions were performed using these pseudoviruses. Three types of pseudoviruses were transfected into HEK293T cells. Following the EMARS reaction, the products were enriched and detected by electrophoresis. EMARS using pseudoviruses derived from DAF-HRP-expressing cells produced strong EMARS products (Fig. S2B; DAF-HRP). In contrast, only trace amounts of the EMARS products were detected when pseudoviruses derived from THY-HRP-expressing cells were used (Fig. S2B; THY-HRP). Subsequent western blot analysis of the post-electrophoresis gel, using an anti-fluorescein antibody, confirmed the presence of fluorescein-labeled proteins, specifically in samples from pseudoviruses generated from DAF-HRP-expressing cells (Fig. S2C).

### Production and characterization of pseudoviruses generated by one-step method

**Fig 1:**
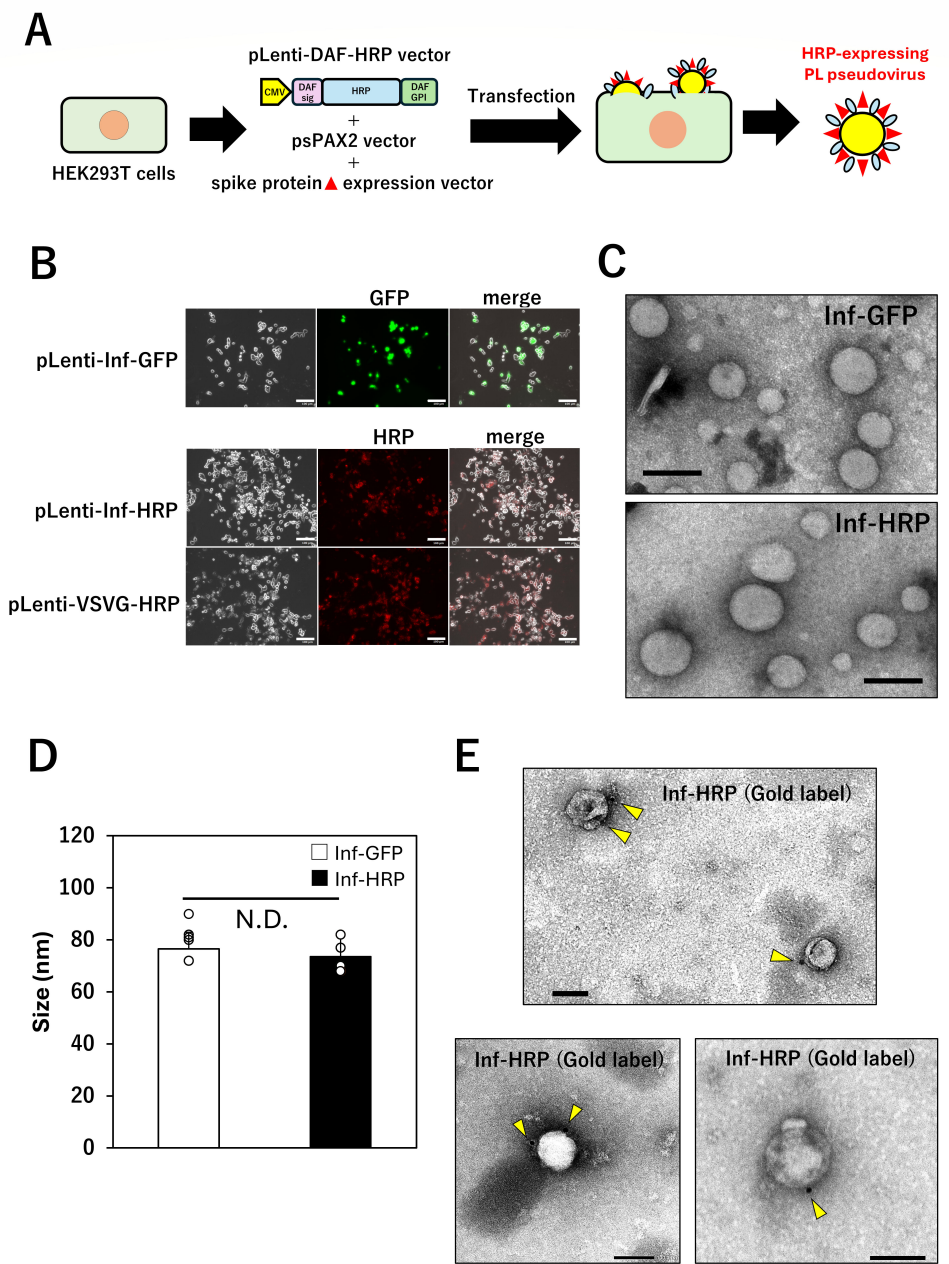
Generation of HRP-expressing pseudovirus. (**A**) Schematic illustration of the procedure for generating pseudoviruses (one-step method). For the production of HRP-expressing pseudoviruses in the one-step method, HEK293T cells (a standard lentiviral packaging cell line) were co-transfected with pLenti-DAF-HRP, viral genes, and the spike protein expression vector of the virus under investigation. (**B**) Detection of HRP expressions in HEK293T cells after viral vector transduction. Following co-transfection of HEK293T cells with the above vectors, the cells were fixed after virus production and stained with an anti-HRP antibody for observation by fluorescence microscopy. Two independent experiments were performed. As a positive control, cells transfected with the pLenti-GFP and virus vector to produce pseudovirus were prepared, and GFP expression was similarly observed. The white bar indicates 100 μm. Two independent rounds of virus production and measurement were performed. (**C–E**) Morphological observation of pseudoviruses using transmission electron microscopy (TEM). The supernatant containing Inf-GFP (upper panel in [**C**]) and Inf-HRP (lower panel in [**C**]) was fixed and observed using TEM. Scale bar, 100 nm. (**D**) Comparison of the diameters of 10 Inf-GFP and 10 Inf-HRP pseudovirus particles. A two-sample Student’s *t*-test was performed, and no significant difference was observed between the two groups. (**E**) Inf-HRP virus produced in the same manner was treated with anti-HRP gold colloid (12 nm: yellow arrowheads) antibody and then observed by TEM. Four individual virus particles with gold colloid labeling are shown in (**E**). In addition, six of the particles are shown in Fig. S3. Scale bar, 100 nm.

**Fig 2:**
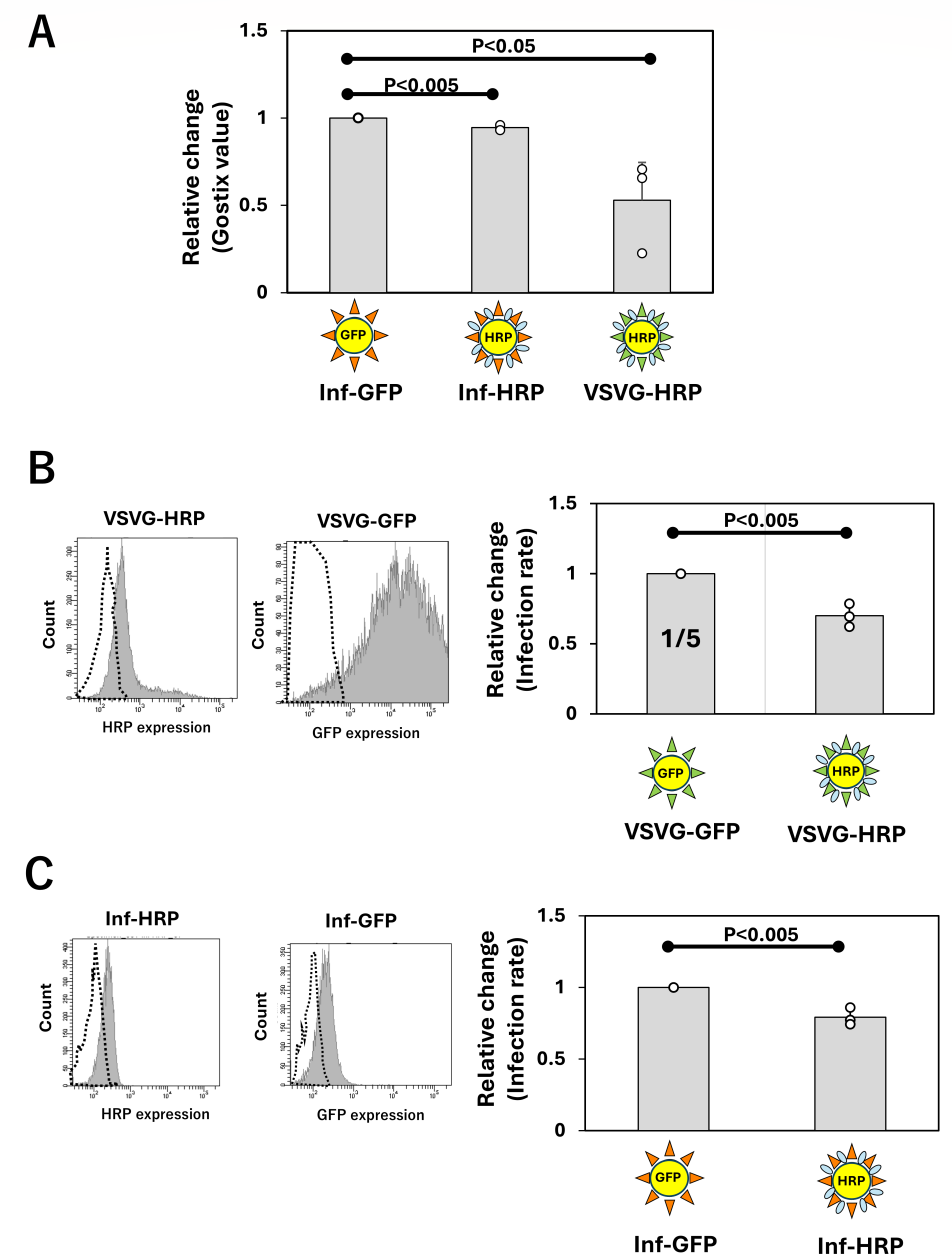
Evaluation of pseudovirus production efficiency and infectivity. (**A**) Comparison of the production efficiency of each pseudovirus using Gostix. Culture supernatants containing each virus (Inf-GFP, Inf-HRP, and VSVG-HRP) were applied to Gostix as described in Materials and Methods, and the detected positive bands were measured using the Gostix app to calculate the Gostix value. The Gostix values of HRP-expressing viruses (Inf-HRP and VSVG-HRP) were expressed as relative values using Inf-GFP as the control. For each virus, three independent rounds of virus production and measurement were performed. Statistical significance between samples was defined as *P* < 0.05. (**B and C**) Evaluation of infection rates for each pseudovirus. A549 cells were treated with the produced VSV-G (**B**) and influenza (**C**) pseudoviruses, and infection rates were assessed based on HRP and GFP expression levels in A549 cells post-infection (Fig. S4). For each virus, three independent rounds of virus production and measurement were performed. The infection rates of VSVG-HRP (**B**) and Inf-HRP (**C**) viruses were expressed as relative values using VSVG-GFP and Inf-GFP as the control, respectively. Statistical significance between samples was defined as *P* < 0.05.

The above HRP-stably expressing packaging cells exhibited weaker adhesion and slower proliferation than standard HEK293T cells (15), posing challenges for stable viral production. Therefore, instead of establishing HRP-stably expressing packaging cell lines, we attempted to generate HRP-expressing pseudoviruses by simultaneously co-transfecting HRP and relevant viral genes directly into HEK293T cells (one-step method; Fig. 1A). In this case, the pLenti-DAF-HRP vector has two roles: (i) pLenti-DAF-HRP vector alone induces DAF-HRP expression in HEK293T cells, and (ii) pLenti-DAF-HRP vector simultaneously plays a role in producing pseudovirus that has the DAF-HRP gene in the virus genome, resulting in the ability to express DAF-HRP in infected cells. HRP expression in packaging cells following transfection was assessed by fluorescence staining. We examined three types of HEK293T cells: those co-transfected with viral vectors expressing both GFP and Influenza virus (PR8) HA as controls and those co-transfected with DAF-HRP together with either VSV-G spike protein or influenza virus (PR8) HA viral vectors. HRP expression was detected in both DAF-HRP-expressing packaging cell types (Fig. 1B; pLenti-Inf-HRP and pLenti-VSV-G-HRP). The expression efficacy seemed to be similar to that of GFP in control cells, which produced the GFP-influenza virus (Fig. 1B). To morphologically assess HRP expression on DAF-HRP-expressing pseudoviruses, TEM was used. When comparing DAF-HRP-expressing and GFP-expressing influenza pseudoviruses (Inf-HRP and Inf-GFP) using TEM, no morphological differences were observed (Fig. 1C). We also compared the diameters of 10 Inf-GFP and 10 Inf-HRP pseudovirus particles, and no significant difference was observed between them (Fig. 1D). Furthermore, upon staining HRP on the surface of DAF-HRP-expressing influenza pseudoviruses with gold-colloid-conjugated anti-HRP antibodies, virions with bound gold colloids were detected on the viral surface; it was consistently observed that each particle displayed no more than two gold colloids (Fig. 1E; Fig. S3). To evaluate the efficiency of DAF-HRP virus production, viral production was compared using the Lenti-X GoStix. The production of GFP-expressing and HRP-expressing influenza viruses was assessed, revealing a slight tendency for lower production of HRP-expressing influenza virus compared to that of GFP (Inf-GFP vs Inf-HRP; Fig. 2A). In contrast, the production of HRP-expressing VSV-G virus was significantly reduced compared to that of both influenza pseudoviruses (Inf-HRP; Fig. 2A).

FACS was performed to quantitatively compare viral infectivity. A549 cells were infected with each pseudovirus, and HRP expression levels in cells treated with HRP-DAF-expressing pseudovirus and GFP expression levels in cells treated with GFP-expressing pseudovirus were measured as indicators of infectivity. Uninfected cells served as negative controls, and GFP or HRP expression levels in infected cells were quantified using flow cytometry. The ratio between infected and whole cells, including uninfected cells, was determined based on analysis of the histogram data as the infection rate (Fig. S4). When comparing HRP-expressing VSV-G with GFP-expressing VSV-G, the titer of GFP-expressing VSV-G was clearly higher; therefore, the volume of the GFP-expressing VSV-G viral solution was reduced to one-fifth of the volume (considering that the production capacity of HRP-expressing VSV-G was half, the actual ratio of viral particles applied to the cells was estimated to be approximately 1:2.5). Following infection, the cells were analyzed using FACS, and as shown in Fig. S4, the infection efficiency of HRP-expressing VSV-G was approximately 70% that of GFP-expressing VSV-G (Fig. 2B). We compared HRP-expressing influenza with GFP-expressing influenza. After infection, the cells were analyzed by FACS, and as calculated in the same way as VSV-G, it was found that the infection efficiency of HRP-expressing influenza was approximately 80% that of GFP-expressing influenza (Fig. 2C).

### Pseudovirus-mediated EMARS reaction

**Fig 3:**
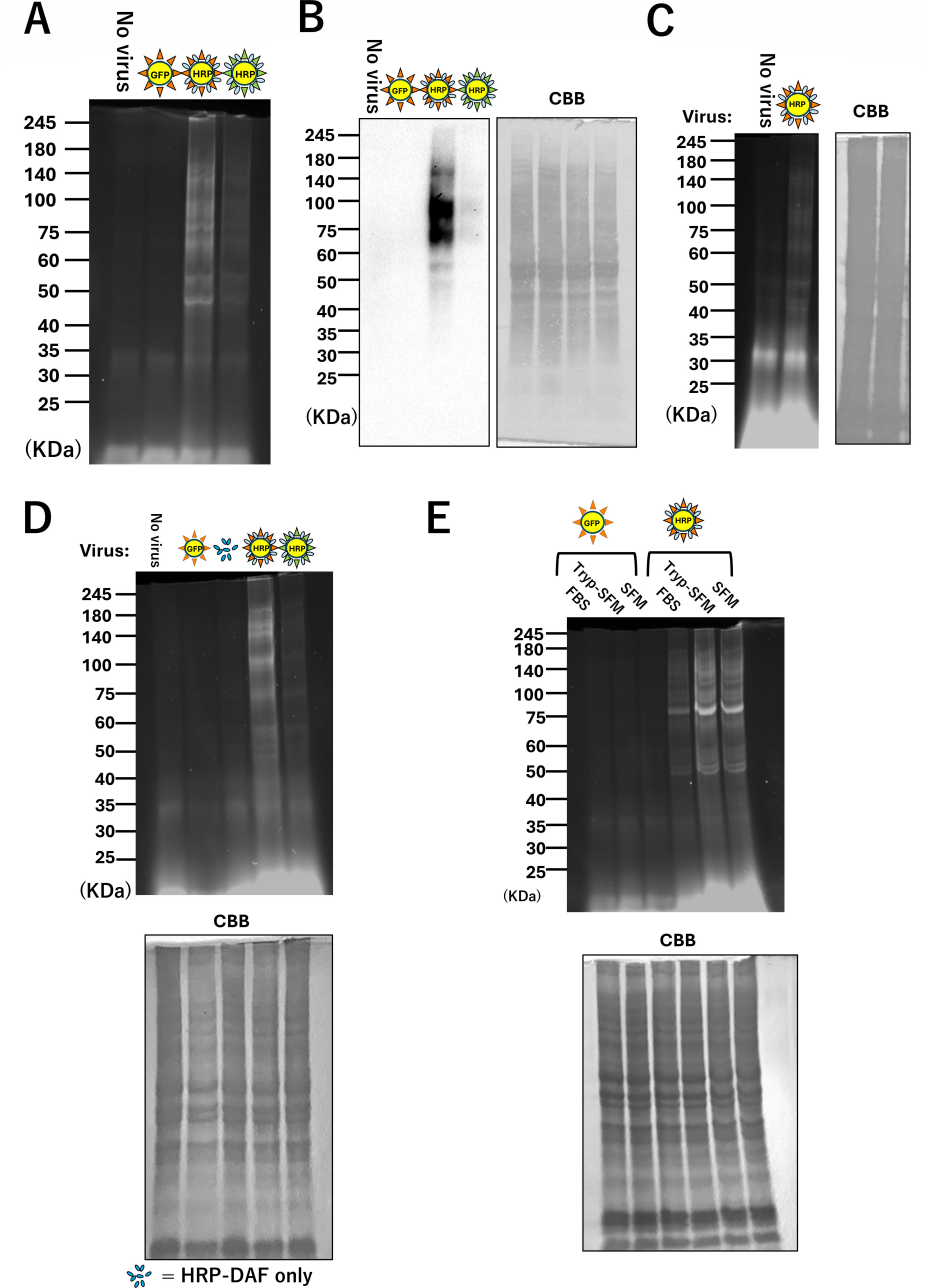
Pseudovirus-mediated EMARS reaction. (**A and B**) SDS-PAGE and western blot analysis of EMARS products. HEK293T cells seeded in the same six-well dish were transfected with each pseudovirus vector set (Inf(PR8)-GFP, Inf(PR8)-HRP, and VSVG-HRP) using the one-step method. Following culture, an equivalent amount of virus-containing culture supernatant from each virus was added to the cells. After thorough washing, FT reagent was added to initiate the EMARS reaction. The labeled molecules were then analyzed by SDS-PAGE as described in Materials and Methods (**A**). The gel was subsequently applied to western blot analysis with anti-fluorescein antibody (**B**). Two independent experiments were performed. CBB staining was used as a loading control. (**C**) SDS-PAGE analysis of EMARS products using Inf-HRP (PR8) pseudovirus purified with a commercially available kit. The pseudovirus in supernatant was purified using the Lenti-X Maxi Purification Kit. CBB staining was used as a loading control. Two independent experiments were performed. (**D**) SDS-PAGE analysis of EMARS products using culture supernatants of HEK293T cells transfected only pLenti-DAF-HRP. EMARS was performed using the culture supernatant of HEK293T cells that expressed only HRP-DAF via transfection with pLenti-DAF-HRP alone in which viral particle formation does not occur. This experiment was performed to determine whether non-viral components are involved in the EMARS reaction. Two independent experiments were performed. (**E**) Each influenza pseudovirus (Inf-HRP and Inf-GFP) was concentrated, resuspended in serum-free medium (SFM), and processed for EMARS reaction either with trypsin (Tryp-SFM) or without trypsin (SFM) treatment. Pseudovirus suspended in standard serum-containing medium (FBS) was also included as a control. Two independent experiments were performed.

The EMARS reaction was successfully induced using pseudoviruses produced by a two-step method (Fig. S2). Therefore, we performed EMARS reactions with pseudoviruses produced using a one-step method. A549 cells were treated with DAF-HRP pseudoviruses carrying either VSV-G or influenza HA (PR8), and after the EMARS reaction, the EMARS products were analyzed by SDS-PAGE. No significant EMARS products were detected when the cells were not treated with any virus, or when treated with GFP-expressing pseudoviruses as negative controls (Fig. 3A). In contrast, obvious or moderate EMARS product bands were observed when the cells were treated with DAF-HRP pseudoviruses carrying either influenza HA or VSV-G (Fig. 3A). Western blot analysis revealed fluorescein-labeled proteins in the same lanes (Fig. 3B). We also performed the EMARS reaction using purified pseudoviruses from the culture medium containing DAF-HRP pseudoviruses carrying influenza HA using a commercially available kit. Although the EMARS reaction was relatively weak, the EMARS products were still detected (Fig. 3C). This indicates that viruses purified using such kits can be used for EMARS. To further clarify whether the viral particles of influenza HA-bearing DAF-HRP pseudoviruses were involved in the EMARS reaction, we modified the one-step method for virus production. Specifically, we omitted the transfection of both the psPAX2 packaging plasmid and the spike protein expression vector, which are essential components of viral packaging, and transfected only the pLenti-DAF-HRP plasmid. No significant EMARS products were detected under these conditions (Fig. 3D). This result confirmed that functional pseudoviral particles requiring proper packaging and spike protein incorporation are indispensable for the EMARS reaction. In addition, as efficient experimental infection by the influenza virus is typically achieved by inducing HA cleavage through trypsin treatment (26), we compared the EMARS products between TPCK-trypsin-treated and untreated pseudoviruses, both solubilized in serum-free medium. No significant difference was observed in the EMARS products with or without trypsin treatment, indicating that trypsin treatment of PL pseudovirus at least does not affect pseudovirus attachment to host cells (Fig. 3E).

### Comparison of EMARS using recombinant spike protein or pseudovirus

In previous studies, EMARS analysis was performed using the recombinant spike protein of SARS-CoV-2 (23). In the present study, to compare the results with those obtained using the newly developed pseudovirus, both the recombinant spike protein and pseudovirus were employed in parallel to compare their EMARS products. Host cells used for this comparison were HeLa-A-T cells expressing ACE2 and TMPRSS2. We examined whether the SARS-CoV-2 spike protein binds to HeLa-A-T cells. FACS analysis revealed that the spike protein bound to these cells (Fig. S5A; *SARS spike*). However, ACE2 expression was low (Fig. S5A; *ACE2*), and additional experiments were performed. Immunocytochemistry of ACE2 staining with an anti-ACE2 antibody showed strong positivity (Fig. S5B; *ACE2*) compared to the SARS spike (Fig. S5B; *SARS spike*). Since cell staining was performed after fixation, it is likely that the antibody used recognized fixed ACE2 more efficiently, whereas ACE2 detection by FACS without fixation resulted in weaker binding signals. We performed the EMARS reaction using both recombinant spike proteins and pseudovirus and detected the EMARS products by SDS-PAGE. The results showed that the band profiles obtained from the two systems were similar; however, the quantity of EMARS products appeared to be lower with the pseudovirus than with the recombinant spike proteins (Fig. S5C). This observation suggests that although both methods can yield comparable patterns of protein labeling, the efficiency of the EMARS reaction may differ depending on the EMARS probe.

### Differences in EMARS products depend on viral spike proteins and influenza HA

**Fig 4:**
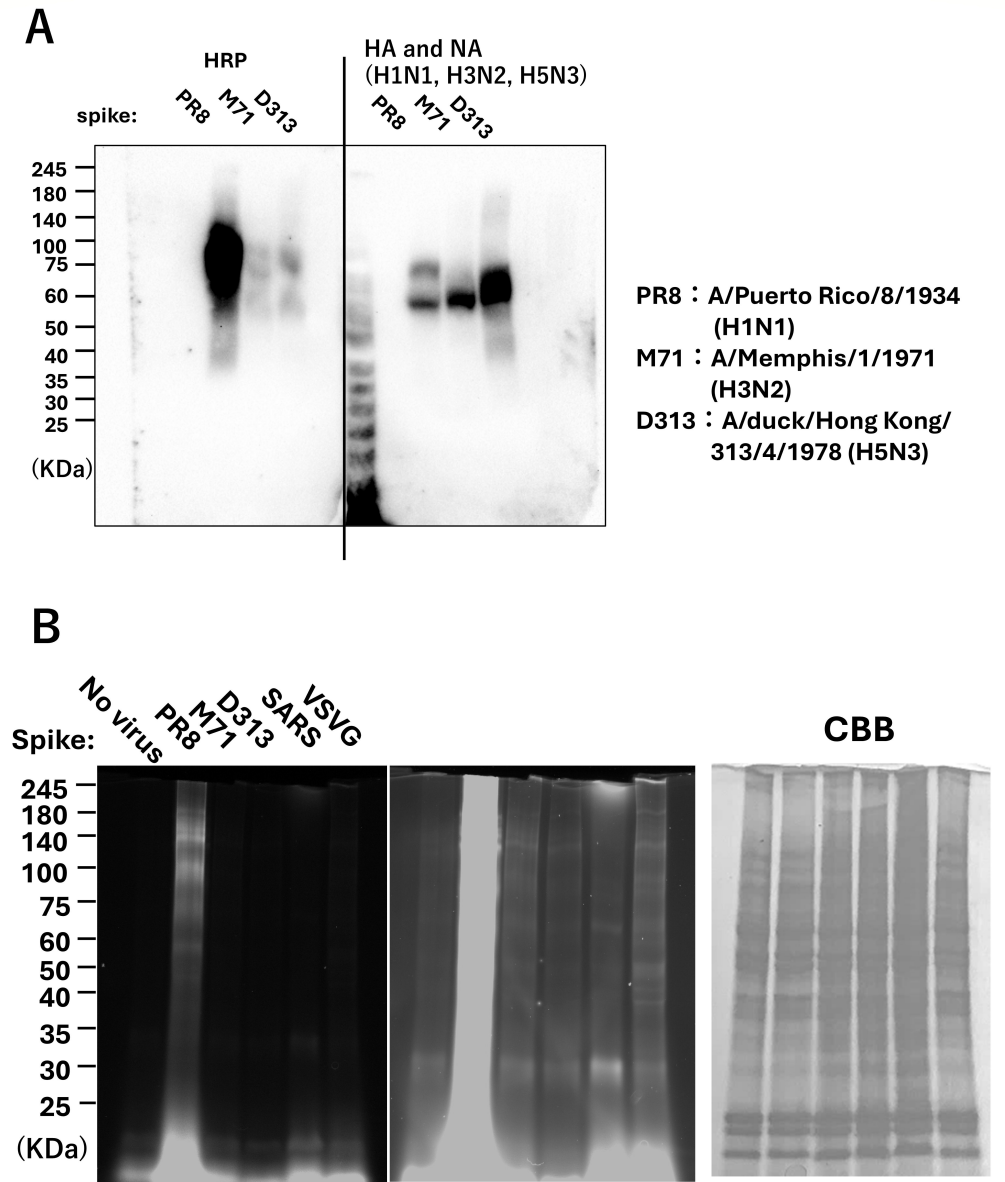
Analysis of EMARS products based on differences in HA and spike proteins. (**A**) Confirmation of HA and HRP expression in each influenza pseudovirus strain. PR8, M71, and D313 influenza pseudovirus strains were concentrated and subjected to western blot analysis. The membranes were stained using anti-HRP antibody (left panel) and anti-HA antibodies (right panel; a mixture of anti-H1N1, anti-H3N2, and anti-H5N3 antibodies) as described in Materials and Methods. Two independent experiments were performed. (**B**) Detection of EMARS products using pseudoviruses carrying each HA and spike protein by EMARS. EMARS was performed using culture supernatants containing the above-mentioned PR8, M71, and D313 influenza HA pseudoviruses, as well as viruses carrying SARS-CoV-2 and VSV-G spike proteins. The EMARS products were analyzed by SDS-PAGE. The center column is an overexposed version of the data on the left column, with the detection sensitivity for samples other than PR8 increased. Two independent experiments were performed. CBB staining was used as a loading control.

To investigate whether differences in EMARS products arise among pseudoviruses bearing different spike proteins and influenza HA, we produced pseudoviruses carrying three types of influenza HA as well as spike proteins from SARS-CoV-2 and VSV-G, using a one-step method and then performed the EMARS reaction. First, we generated pseudoviruses carrying each of the three types of influenza HA and examined the expression of viral HRP and HA proteins using western blotting. Among the PR8, M71, and D313 viruses, D313 HA was expressed at higher levels than the other viruses (Fig. 4A; HA). In contrast, HRP expression was markedly higher in the pseudoviruses carrying PR8 (Fig. 4A; HRP). Therefore, these results suggest that the PR8 pseudovirus may contain a greater amount of HRP per virion than other viruses.

In addition to these three types of influenza pseudoviruses, we treated A549 cells and HeLa-A-T cells (for SARS-CoV-2) with a total of five pseudoviruses: those carrying PR8HA, M71HA, D313HA, VSV-G, and SARS-CoV-2 spike proteins, and performed the EMARS reaction. The EMARS products were analyzed using electrophoresis. Many EMARS products were detected in the EMARS reaction with the PR8 pseudovirus (Fig. 4B; PR8). In the cases of M71, D313, and VSV-G pseudoviruses, fewer EMARS products were observed compared to PR8; however, some bands were detected, and the band patterns appeared to differ depending on the viral species (Fig. 4B; PR8). Because the host cell type for SARS-CoV-2 was different, a direct comparison may not be appropriate.

### EMARS and proteomic analysis showed the candidate HCAF

The EMARS reactions in A549 cells by three types of pseudoviruses (DAF-HRP pseudovirus carrying VSV-G and Influenza PR8 with GFP pseudovirus carrying Influenza PR8 as a negative control virus) were performed, and then purified and enriched biotinylated EMARS products, followed by proteome analysis using MS (performed in duplicate; Tables S1 to S5).

For shotgun analysis using mass spectrometry, biotinylated peptides were enriched using NeutrAvidin beads. From the EMARS samples prepared using three types of pseudoviruses, a total of 216 proteins (in the first MS analysis) and 317 proteins (in the second MS analysis) were identified. Among them, 26 (first analysis) and 39 (second analysis) proteins were directly labeled with biotinyl tyramide. In addition, the proteins that were scarcely detected in the negative control GFP-expressing pseudovirus sample (with an abundance ratio ≥ 100 (i.e., detected at 100-fold or greater levels compared to the negative control), according to the Proteome Discoverer ver. 3.1 software) were 19 (first analysis) and 27 (second analysis). Of these, 18 molecules were detected in both experiments, all of which appeared in both the influenza PR8 and VSV-G samples. However, comparison of the abundance ratios revealed that these proteins were detected at approximately fivefold to comparable levels in the Inf-HRP samples relative to the VSV-G samples (Table 1).

**TABLE 1:** Selected candidates for proximal membrane proteins around attached influenza (Inf) and VSV-G pseudovirus to A549 cells

Accession no.	Proteins	Inf/VSV-G
B4DUF2	cDNA FLJ50227, moderately similar to Complement decay-accelerating factor	5.645
P15529	Membrane cofactor protein	4.15
Q59ER8	Leucine-rich repeat-containing G-protein coupled receptor 4	3.158
P78310	Coxsackievirus and adenovirus receptor	3.134
O14786	Neuropilin-1	3.108
Q8TCZ2	CD99 antigen-like protein 2	3.039
A8K6Q6	CD276 antigen	2.869
O75942	Major prion protein	2.797
Q14126	Desmoglein-2	2.297
Q12860	Contactin-1	2.123
A5YM53	Integrin alpha-V	2.002
Q13740	CD166 antigen	1.835
Q9NZU0	Leucine-rich repeat transmembrane protein FLRT3	1.449
P16070	CD44 antigen	1.432
Q96S97	Myeloid-associated differentiation marker	1.271
Q9UHN6	Cell surface hyaluronidase CEMIP2	1.208
C9JPK5	Integrin beta	1.037
Q9UNN8	Endothelial protein C receptor	1.015

### Virus attachment assay using the candidate HCAF-expressing CHO-K1 cells

**Fig 5:**
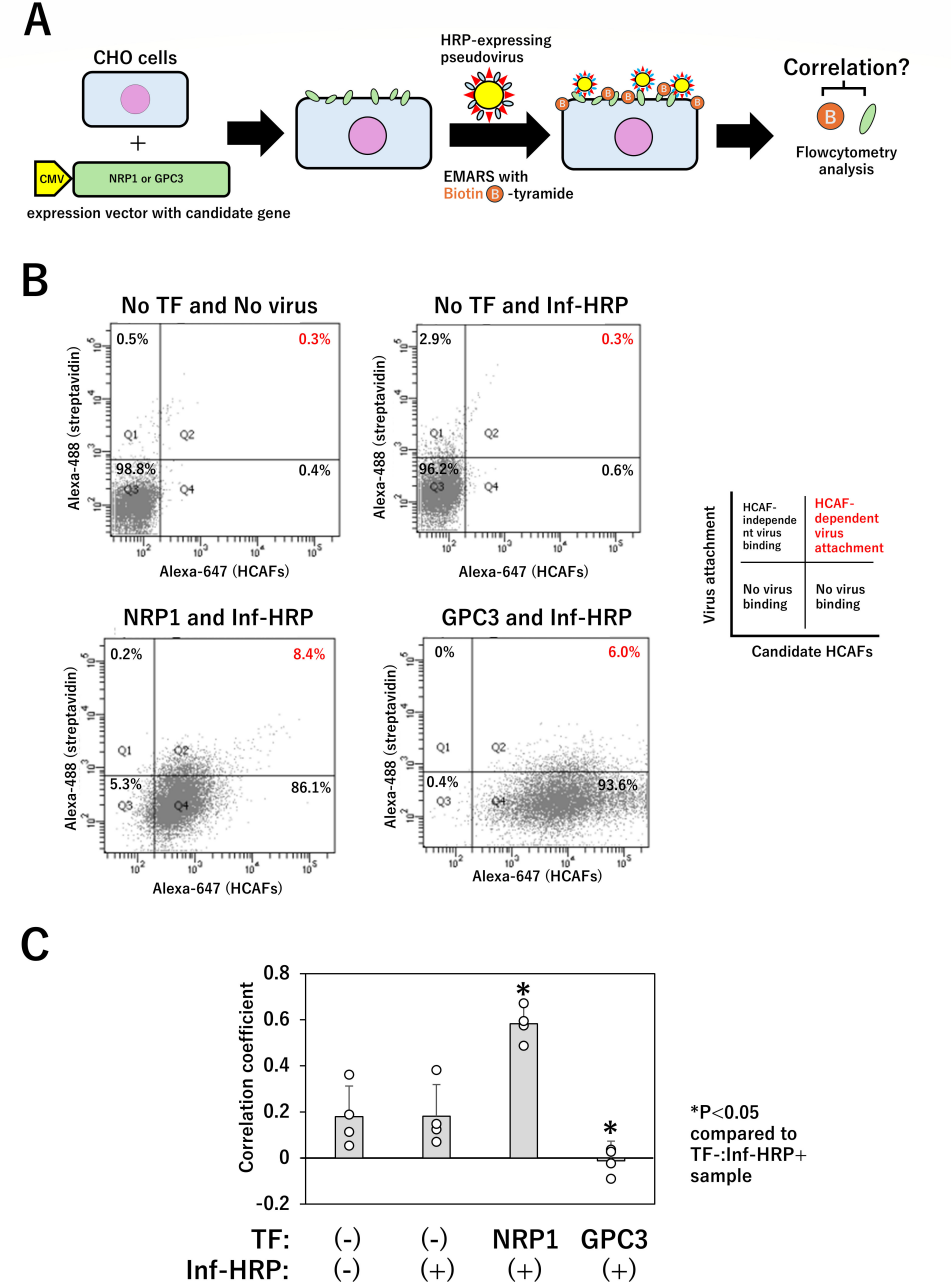
Virus attachment assays for HCAF confirmation. (**A**) Schematic illustration of the procedure for virus attachment assay. CHO-K1 cells were transiently transfected with expression vectors of NRP1 and GPC3, and then treated with Inf (PR8)-HRP pseudovirus followed by EMARS reaction with biotin tyramide as described in Materials and Methods. (**B**) Representative FACS dot plot analysis. Fluorescence intensities of Alexa Fluor 488 and 647 in each cell were measured by FACS, and the results were plotted as dot plots. The vertical axis represents virus attachment (Alexa Fluor 488 fluorescence), and the horizontal axis indicates the expression level of the candidate HCAF molecule (Alexa Fluor 647 fluorescence). The plots were divided into four quadrants (vertical axis: 200 and horizontal axis: 700) to assess viral attachment in CHO-K1 cells expressing candidate HCAFs. Four independent experiments were performed. (**C**) Correlation analysis between candidate molecule expression and virus attachment. The correlation between these two parameters was assessed by calculating correlation coefficients. A statistically significant correlation (*P* < 0.05) was observed across all samples. Furthermore, four independent analyses were performed, and the correlation coefficients from each analysis were compared. *P < 0.05: compared to TF−:Inf-HRP+ sample.

Proteomic analysis identified candidate proteins that are potentially involved in viral attachment to cells. Examination of the molecules listed in Table 1 revealed that many of them have been reported as potential viral receptors, including membrane cofactor protein (CD46), leucine-rich repeat-containing G-protein coupled receptor 4 (LGR4), and coxsackievirus and adenovirus receptor (CAR). CD46 serves as a receptor for measles and herpesviruses (27), LGR4 for the VSV-G virus itself (28), and CAR for adenoviruses (29). Among these, NRP1 was selected for the virus attachment assay because several reports have suggested that NRP1 may function as a common receptor for multiple unrelated viruses (30–35). Glypican-3 (GPC3), a negative control molecule, was previously used as a candidate HCAF for SARS-CoV-2 (23). To eliminate the influence of endogenous human cell membrane proteins, we transiently expressed the three candidate molecules in CHO-K1 cells. These cells were then treated with a pseudovirus, and the attached virus was quantified. To enhance the sensitivity of viral attachment detection, we performed EMARS using biotinyl tyramide on DAF-HRP pseudovirus-treated CHO-K1 cells, with the biotin signal on the cells serving as an indicator of viral attachment (Fig. 5A). After EMARS, CHO-K1 cells were treated with primary antibodies specific to each of the three candidate molecules. The cell samples were subsequently incubated with secondary antibodies conjugated to Alexa Fluor 647 and streptavidin-Alexa Fluor 488. The processed cell samples were analyzed by flow cytometry (FACS), and the fluorescence intensity of individual cells was visualized using dot plots. In this experiment, CHO-K1 cells that were not transfected with candidate molecules and were either treated or untreated with Inf-HRP were used as negative controls (Fig. 5B; “No TF and No virus” or “No TF and Inf-HRP”). Representative plots (Fig. 5B) were divided into four quadrants (vertical axis: 200, horizontal axis: 700; the “No TF and Inf-HRP” plots were treated as the “no attachment” sample) to assess viral attachment in CHO-K1 cells expressing candidate HCAFs. Since there is approximately a 0.3% signal in the second quadrant for “No TF and Inf-HRP,” the 0.3% threshold was used as the negative attachment. Among the four fractions, the fraction where both biotin and the candidate molecule were detected (double positive in the upper-right area; Fig. 5B) was operationally defined as the cell population of the candidate molecule-dependent attachment. Representative data revealed that double-positive plots were slightly increased in CHO-K1 cells expressing NRP1 (8.4% population) compared to negative control cells expressing GPC3 (6.0% population) (Fig. 5B). However, given that these differences were minimal across all four independent experiments, we determined that the correlation between the candidate HCAF expression levels and pseudovirus attachment (cell surface biotin) in individual cells could serve as a primary discriminatory index. This analysis included all the cells (approximately 10,000 cells per experiment) subjected to FACS in each trial. Fluorescence intensities were quantified using FlowJo software, and correlation coefficients were calculated and compared with those of negative control cells (*P* value of correlation coefficients in all experiments was <0.05). The results of four independent experiments indicated that only NRP1-expressing cells showed a significant positive correlation between NRP1 expression and viral attachment, in contrast to a significant negative correlation with GPC3 as a negative control (Fig. 5C).

## DISCUSSION

Pandemic viruses, including newly emerging variants such as avian influenza viruses, will continue to pose a threat, and many pathogenic viruses still lack clearly defined host cell attachment factors (HCAFs) (36). To address this issue, it is important to establish an efficient system for screening HCAF candidates as an initial step in characterizing viral entry mechanisms.

In this study, we developed an experimental platform based on proximity labeling (PL) technology to identify HCAF candidates using pseudotyped viruses. Because viral attachment and entry occur within a short time frame and only a limited number of viral particles are expected to bind to host cells, we considered that a method with rapid reaction initiation and high labeling efficiency would be required. We therefore adopted EMARS, a radical-based PL method employing HRP, which we previously used to identify HCAFs for SARS-CoV-2 with recombinant spike proteins (23). However, the use of recombinant proteins raises concerns about whether they fully recapitulate the properties of authentic viral particles, especially for rapidly mutating pandemic viruses. In contrast, the present system enables HCAF analyses using only the genetic information encoding viral spike or envelope proteins.

To generate pseudoviruses expressing the PL enzyme, we used lentivirus-based vectors and engineered the packaging cells to present HRP on the viral envelope. We achieved this by constructing a fusion protein comprising HRP and a GPI-anchor domain, which enables stable membrane localization (14, 15). A practical advantage of this strategy is that pseudoviruses bearing different viral spike proteins can be produced simply by exchanging the spike expression vector, without time-consuming genome engineering of each target virus. Among the constructs tested, DAF-HRP supported stronger EMARS reactivity than Thy1-HRP, although stable expression of DAF-HRP in HEK293T cells reduced cell proliferation and viability (14, 15). Thus, depending on whether a reporter gene (GFP) is required, either a two-step or one-step production protocol should be selected to balance virus yield and experimental feasibility.

We next compared EMARS using recombinant spike proteins from our previous work with EMARS using pseudoviruses in the present system. As expected from the size difference between virus particles and recombinant spike proteins, EMARS reactivity was lower with pseudoviruses; nonetheless, similar banding patterns were observed. Although reduced labeling efficiency may limit the detection of some HCAFs, we prioritized physiological relevance and therefore regard pseudovirus-based EMARS as a more appropriate initial approach for identifying HCAF candidates.

The impact of DAF-HRP expression on pseudovirus properties varied depending on the viral spike protein. Production efficiency and infectivity of influenza pseudoviruses were only moderately reduced compared with controls, allowing their use for HCAF identification, whereas VSV-G pseudoviruses showed a more pronounced decrease in infectivity. PR8 influenza pseudoviruses carried relatively high amounts of DAF-HRP and yielded abundant EMARS products, while VSV-G pseudoviruses produced fewer EMARS products, consistent with the lower viral production and infectivity. These results indicate that the suitability of each pseudovirus for HCAF labeling should be assessed individually, taking into account both virus production and infection efficiency.

Comparison of EMARS products among different viruses suggested that HCAF profiles may vary between virus species. At the same time, proteomic analyses identified multiple molecules that were shared by influenza and VSV-G pseudoviruses in the same host cells, implying that common HCAFs can also be exploited under certain conditions.

From the set of candidate molecules obtained by mass spectrometry, it is not realistic to perform knockdown or knockout analyses for all candidates, particularly in the context of rapidly emerging pandemic viruses. To address this, we established a simplified screening assay using CHO-K1 cells, in which endogenous human membrane proteins are absent and EMARS enhances the sensitivity of detecting viral attachment without the need for virus-specific antibodies.

By combining EMARS labeling with FACS analysis of double-positive cells and correlation analyses between HCAF expression and viral attachment, we identified NRP1 as a promising HCAF candidate. NRP1-expressing cells showed higher frequencies of double-positive populations than a negative-control molecule, and quantitative analysis across multiple experiments demonstrated a significant correlation between NRP1 expression and influenza pseudovirus attachment. NRP1 has been reported as an HCAF for several viruses, including SARS-CoV-2 (10, 31–36). These findings support the notion that NRP1 can function as a common attachment factor across different enveloped viruses.

This screening and attachment assay for HCAF candidates offers several practical advantages: it is experimentally simple, cost-effective, and can be implemented in general biology laboratories. Nonetheless, several limitations should be acknowledged. First, the generation of HRP-expressing pseudoviruses may be technically demanding for certain viral species, as exemplified by VSV-G. Second, even with the same pseudovirus and host cells, EMARS band patterns can vary among experiments, necessitating repeated analyses for reliable evaluation.

In conclusion, despite these limitations, the present EMARS-based pseudovirus system provides a useful first-line screening tool for HCAF identification, owing to its simplicity, cost-effectiveness, and broad applicability. We anticipate that this approach will contribute to elucidating the attachment and entry mechanisms of a variety of enveloped viruses and to the future development of novel antiviral strategies (23, 36).

## MATERIALS AND METHODS

### Cell culture

HEK293T cells (Lenti-X 293T Cell Line; Takara Bio) and its transfectant cells were cultured in RPMI 1640 medium (Wako Chemicals) supplemented with 10% fetal bovine serum (FBS; GIBCO) at 37°C in a humidified 5% CO₂ atmosphere. HEK293T cells used as packaging cells for viral production were seeded in collagen-coated dishes (Iwaki). A549 human Caucasian lung carcinoma (JCRB0098; Japanese Collection of Research Bioresources Cell Bank, Osaka, Japan) and HeLa cells co-expressing ACE2 and TMPRSS2 (designated as HeLa-A-T; JCRB1835, Japanese Collection of Research Bioresources Cell Bank, Osaka, Japan) were maintained in RPMI 1640 medium supplemented with 5% FBS at 37°C in a humidified 5% CO₂ atmosphere. Chinese hamster ovary (CHO)-K1 cells (IFO50414; Japanese Collection of Research Bioresources Cell Bank, Osaka, Japan) were maintained in Ham’s F12 medium (Wako Chemicals) supplemented with 10% FBS at 37°C in a humidified 5% CO₂ atmosphere.

### Production of packaging cells expressing HRP

The fusion genes of the human codon-optimized-HRP gene and GPI anchor motif with signal sequence of complement decay-accelerating factor (DAF) or Thy1 proteins were cloned into the pLenti CMV Puro DEST vector (AddGene # 17,452) (14, 15). These vectors are hereafter referred to as pLenti-DAF-HRP and pLenti-THY-HRP. HEK293T cells stably expressing HRP were generated by direct transfection with pLenti-DAF-HRP and pLenti-THY-HRP, using typical transfection reagents. However, conventional transient transfection may yield a low HRP expression efficiency, necessitating selection and cloning. To overcome this limitation, we used lentiviral transduction of HEK293T cells to establish a stable cell line. To generate viruses carrying DAF-HRP and THY-HRP genes, 800 ng of this HRP-containing plasmid was co-transfected with 800 ng psPAX2 packaging plasmids (AddGene #12,260) and 800 ng pMD2.G spike protein expression plasmid (AddGene #12,259) into HEK293T cells (in 10 cm dishes and grown to ~80% confluency) using TransIT-2020 reagent (Mirus and Takara Bio) with standard transfection protocols. The treated cells were cultured for 72 h and then supernatant containing viruses was harvested followed by filtering with 0.45 µm filter (Sartorius). One milliliter of the filtered supernatant was added to newly plated HEK293T cells. After 72 h of incubation, puromycin selection was initiated at 2 µg/mL. Positively selected cells were maintained in puromycin-containing medium through successive passages and cryopreserved at −80°C using CultureSure cell preservation solution (Fujifilm Wako). Engineered cells (hereafter referred to as “DAF-HRP packaging cells” or “THY-HRP packaging cells”) were assessed for HRP expression using fluorescence microscopy and fluorescence-activated cell sorting (FACS) (*see* details below). Cells with consistent HRP expression were used as stable HRP-expressed HEK293T packaging cells for pseudovirus production.

### Production of PL pseudovirus

Two distinct methods were employed to generate PL viruses. In the first approach (herein referred to as the two-step method), the packaging cells established by stably expressing HRP in the HEK293T cells described above were applied to viral production. To generate DAF-HRP- or THY-HRP-expressing pseudoviruses (hereafter referred to as “DAF-HRP pseudovirus” or “THY-HRP pseudovirus”) bearing the VSV-G spike protein, HRP-expressing packaging cells were transfected with pLenti CMV GFP Puro (AddGene #17,448), psPAX2 (AddGene), or pMD2.G (AddGene) using standard transfection protocols. The resulting supernatants, containing HRP and GFP-expressing VSV-G viruses, were collected for downstream applications.

In the second approach (herein referred to as one-step method), to generate HRP-expressing pseudoviruses without utilizing pre-established HRP-expressing packaging cells, we directly co-transfected HEK293T cells with the following components: the pLenti-DAF-HRP plasmid constructed as described previously, psPAX2, and a spike protein expression vector of interest. For VSV-G pseudoviruses, pMD2.G was used for spike protein expression, following the procedures described above. To produce HRP-expressing pseudoviruses displaying the SARS-CoV-2 spike protein, the pPACK-SPIKE SARS-CoV-2 “S” Pseudotype Lentivector Packaging Mix (Wuhan-Hu-1; CVD19-500A-1; SBI System Biosciences) and pLenti-DAF-HRP but not psPAX2. For influenza pseudoviruses, the hemagglutinin (HA) genes from three different influenza strains, A/Puerto Rico/8/1934 (H1N1:PR8), A/Memphis/1/1971 (H3N2:M71), and A/duck/Hong Kong/313/4/1978 (H5N3:D313) were cloned respectively into the pCAGGS expression vector and used for co-transfection with the pLenti-DAF-HRP and psPAX2. Under all conditions, 800 ng of each plasmid was used per 10 cm dish, and 200 ng per plasmid was used for transfection in 6-well plates. Transfection was performed using the standard TransIT-2020 protocol. Viral supernatants were harvested 48-72 h post-transfection, filtered through 0.45 µm membranes (S7598FXOSK; Sartorius), and used for subsequent experiments. In some experiments, the pseudovirus in the supernatant was purified using a Lenti-X Maxi Purification Kit (631,233; Takara Bio) according to the manufacturer’s instructions.

### Flow cytometry

Each cell was stained with i) Rhodamine-conjugate anti-HRP antibody (123-295-021; Jackson ImmunoResearch; 5 µg/mL 0.5% BSA-PBS) at room temperature for 20 min, ii) anti-ACE2 antibody (PAB886Hu01; CLOUD-CLONE, 5 µg/mL 0.5% BSA-PBS) at room temperature for 20 min followed by anti-rabbit IgG Alexa Fluor 488 (ab150077; Abcam; 10 µg/mL 0.5% BSA-PBS) at room temperature for 20 min, or iii) recombinant SARS-CoV-2 spike protein (S1-RBD; 40592-V05H; S1-RBD-mouse Fc, Sino Biological) at room temperature for 20 min followed by the secondary anti-mouse IgG Alexa Fluor 488 antibody (A-11001; Thermo Fisher Scientific; 5 µg/mL 0.5% BSA-PBS). After washing with PBS, the treated cells were analyzed using a FACS Canto II flow cytometer (BD Biosciences) to determine the expression of each molecule or the binding of RGD spikes.

### Immunocytochemistry

For the confirmation of HRP expression in DAF-HRP packaging cells and THY-HRP packaging cells, stable HRP-expressing HEK293T packaging cells were detached using trypsin-EDTA (Wako), transferred to plastic tube, then stained with a goat anti-HRP primary antibody (123-005-0211; Jackson ImmunoResearch) at room temperature for 30 min followed by Alexa Fluor 488-conjugated donkey anti-goat IgG secondary antibody (ab150129; Abcam; 10 µg/mL 0.5% BSA-PBS). After washing with PBS, the treated cells were transferred to 35-mm glass-bottom dishes (627,870; Greiner). HeLa-A-T cells were stained with an anti-ACE2 antibody (PAB886Hu01; CLOUD-CLONE, 5 µg/mL 0.5% BSA-PBS) at room temperature for 20 min followed by anti-rabbit IgG Alexa Fluor 488 (ab150077; Abcam; 10 µg/mL 0.5% BSA-PBS) at room temperature for 20 min, or recombinant SARS-CoV-2 spike protein (S1-RBD; 40592-V05H; S1-RBD-mouse Fc, Sino Biological) at room temperature for 20 min followed by the secondary antibody anti-mouse IgG Alexa Fluor 488 (A-11001; Thermo Fisher Scientific; 5 µg/mL 0.5% BSA-PBS). HEK293T cells directly transfected with pLenti-DAF-HRP and each virus vector were fixed with 4% paraformaldehyde (09154-85; Nacalai Tesque) and then stained with Rhodamine-conjugate anti-HRP antibody (123-295-021; Jackson ImmunoResearch; 5 µg/mL 0.5% BSA-PBS) at room temperature for 30 min. These stained samples and the virus-infected cells expressing GFP were observed with a fluorescent microscope BZ-700 (Keyence) or EVOS FLoid Cell Imaging Station (Thermo Fisher Scientific). Raw images, including differential interference contrast images, were captured under identical settings for the same experiments and then exported as TIFF or JPEG files.

### Assays of virus production and infection

In the virus production assay, the Lenti-X GoStix Plus (631281; Clontech and Takara Bio) was used to measure the amount of virus produced. A 20 μL aliquot of packaging cell culture supernatant (containing virus virion) was applied to the Lenti-X GoStix Plus stick, followed by the addition of 80 μL of the supplied buffer. The intensity of the virus-positive band was quantified using the GoStix Plus application (Clontech and Takara Bio). The amount of virus was expressed as the “GoStix value” obtained from the GoStix Plus app.

In the virus infection assay, each virus supernatant obtained from the virus production method described above was filtered using a 0.45 µm filter (Sartorius). Lenti-X Concentrator (Takara Bio) was added to the filtered supernatant at a 1:3 (vol/vol) ratio and then incubated overnight at 4°C to enrich viral particles. The incubated sample was centrifuged at 1,500 × *g* for 45 min to pellet the virus. The supernatant was discarded, and the pellet was resuspended in 300 μL of serum-free medium (SFM; ASF104; Ajinomoto) with thorough mixing. The resuspended viral solution was added to one well containing A549 cells seeded in a 12-well plate. For GFP-expressing VSV-G pseudoviruses, a 1/5 volume (60 μL) was used because of high titers. Treated cells were cultured at 37°C for 48 h. After infection, the A549 cells were washed three times with PBS, detached using trypsin, and harvested. To assess the infectivity of the DAF-HRP pseudoviruses, we used HRP expression as an infection marker in infected host cells because they express HRP instead of GFP. These HRP-expressing A549 host cells were stained with a rhodamine-conjugated anti-HRP antibody (123-295-021; Jackson ImmunoResearch; 5 µg/mL in 0.5% BSA-PBS) as described above. Infected A549 host cells expressing GFP or HRP were analyzed by flow cytometry, as described above. Non-virus-treated A549 cells were used as negative controls. For convenience, the extent of infection was quantified using FACS data as follows: the histogram images obtained from FACS analysis were processed using the image analysis software Fiji (37). For each virus-infected cell population, the area of the histogram that did not overlap with the histogram of the non-virus-treated A549 cells (measured using Fiji software) was defined as infected cells. The infection rate of each virus was calculated as the ratio of the area of infected cells to the total number of cells.

### EMARS reaction

The EMARS reaction was performed as previously described (12, 38). In this study, EMARS was performed using two types of EMARS reagents. One was the conventional fluorescein-conjugated tyramide (FT) reagent, which was used for the electrophoretic analysis of EMARS products. The other, the biotinyl tyramide (BT) reagent, was employed for the subsequent proteomic analysis and the virus attachment assay described below. In the case of the electrophoretic analysis, each cell was cultured in 10 cm, 6 cm, or six-well plastic culture dishes (TPP) until approximately 80–90% confluent and they were subsequently treated with HRP-conjugated probes (EMARS probe) or HRP-expressing pseudovirus. For EMARS using SARS-CoV-2 spike protein, the spike protein (0.5 µg/mL 0.5% BSA-PBS) was treated at room temperature for 30 min. After washing with PBS three times, the cells were treated with HRP-conjugated anti-mouse IgG (W402B; Promega; 0.25 µg/mL 0.5% BSA-PBS) at room temperature for 30 min. After washing with PBS, the treated cells were then incubated with 0.05 mM FT (14) containing 0.0075% H_2_O_2_ in PBS at room temperature for 20 min in the dark.

Purified and enriched pseudovirus or supernatant containing pseudovirus was added to each cell and incubated at room temperature for 2–30 min (depending on the experiment). For trypsinization of influenza viruses, 1 μL TPCK-trypsin solution (2023; Thermo Scientific; 1 mg/mL) was added to the virus in SFM solution, followed by incubation at 37°C for 30 min. After treatment with these viruses, the host cells were then incubated with 0.05 mM FT or BT with 0.0075% H_2_O_2_ in PBS at room temperature for 20 min in the dark. Host cells subjected to EMARS were homogenized with 100 mM Tris-HCl (pH 7.4) using a 22 G syringe needle to rupture the plasma membrane fractions, centrifuged at 20,000 × *g* for 15 min to precipitate the plasma membrane fractions, and used for subsequent experiments.

### Detection of EMARS products (fluorescein-labeled proteins) in gels

Sodium dodecyl sulfate-polyacrylamide gel electrophoresis (SDS-PAGE) was performed to confirm the presence of EMARS products. The precipitates containing the plasma membrane fraction were resuspended by reducing the SDS sample buffer (09499-14; Nacalai Tesque), sonicated for approximately 5 s, and then heated at 100°C for 5 min. After cooling, each sample was resolved by 8% SDS-PAGE using Rapid Running Buffer Solution (12981-74; Nacalai Tesque). Protein Ladder One Plus, Triple-color (19593-25; Nacalai Tesque) was used as the molecular weight marker. The gel was analyzed using a ChemiDoc Touch (Bio-Rad; equipped with fluorescein filter) to detect fluorescein-labeled EMARS products. For loading controls, SDS-PAGE gels were stained with Coomassie brilliant blue solution, if necessary.

### Preparation of biotinylated EMARS products for mass spectrometry analysis

The sample pellets treated with EMARS using BT reagent (see “EMARS reaction,” above) were extracted with 500 µL chloroform:methanol (2:1, vol/vol) and gently mixed for 5 min at room temperature, followed by the addition of 500 µL water and further mixing for 5 min. The mixture was centrifuged at 15,000 rpm for 1 min at room temperature to induce phase separation, and both the upper aqueous phase and the lower organic phase were carefully removed. The remaining pellet was washed two times with 1 mL 40% methanol, each time followed by centrifugation at 15,000 rpm for 1 min, and the supernatant was discarded. The washed pellet was then resuspended in 100 µL PTS buffer (127-0710; Fujifilm Wako; 100 mM Tris-HCl, pH 9.0, 12 mM sodium deoxycholate, 12 mM sodium lauroyl sarcosinate, and protease inhibitor cocktail) and mixed thoroughly. The samples were treated with 10 mM dithiothreitol (DTT; Nacalai Tesque) at 50°C for 30 min and subsequently alkylated with 50 mM iodoacetamide (Fujifilm Wako) in 50 mM ammonium bicarbonate buffer at 37°C for 1 h in the dark. The sample volume was then adjusted fivefold with 400 µL 50 mM ammonium bicarbonate. Proteins were first digested with Lys-C (121-05063; Fujifilm Wako; 0.25 µg/µL) by adding 2 µL and incubating at 37°C for 3 h, followed by digestion with sequencing-grade modified trypsin (V5280; Trypsin Gold, MS grade; Promega; 1 µg/µL) by adding 2 µL and incubating overnight at 37°C.

For enrichment of biotinylated peptides, 20 µL Sera-Mag SpeedBeads NeutrAvidin-Coated Magnetic Particles (78152104011150; Cytiva) were placed on a magnetic rack and washed once with 1 mL 1× TBS. The digested peptide solution was mixed with 500 µL 1× TBS, centrifuged at 15,000 rpm for 10 min at room temperature to remove insoluble debris, and the supernatant was transferred to a new tube. The supernatant was then added to the pre-washed NeutrAvidin beads and rotated at room temperature for 1 h to capture biotinylated peptides. After incubation, the beads were collected using a magnetic rack and washed five times with 1 ml 1× TBS. Bound peptides were eluted by adding 100 µL 5% acetonitrile/0.1% trifluoroacetic acid (TFA), resuspending thoroughly, and heating at 100°C for 5 min, after which the supernatant containing the eluted peptides was carefully collected. For peptide purification, the eluted sample was applied to a GL-Tip SDB (7820-11200; GL Science) according to the manufacturer’s instructions. Peptides were eluted into a fresh 1.5 mL tube with 20 µL 70% acetonitrile/0.1% TFA by centrifugation at 3,000 rpm for 2 min. The eluates were concentrated in a vacuum evaporator for approximately 15 min until a residual volume of 3–5 µL remained, and finally, 1–3 µL of 5% acetonitrile/0.1% TFA was added to adjust the volume for LC–MS/MS analysis.

### Proteomic analysis of EMARS products using mass spectrometry analysis

Proteomic analysis was performed using nano-liquid chromatography-electrospray ionization mass spectrometry (nano-LC-ESI-MS/MS). The prepared samples were injected into a Vanquish Neo UHPLC system (Thermo Fisher Scientific). MS analysis was performed using a Thermo Scientific Orbitrap Eclipse Tribrid Mass Spectrometer equipped with a nano-ESI source. A NIKKYO nano HPLC capillary column (3 μm C18, 75 μm I.D. × 120 mm; Nikkyo Technos) was used, and this was preceded by a C18 PepMap100 column (300 μm I.D × 5 mm; Thermo Fisher Scientific). The peptides were eluted from the column using a 40–35% acetonitrile gradient over 90 min. The eluted peptides were directly electrosprayed onto the spectrometer and subjected to precursor and data-dependent MS/MS analysis (DDA).

Precursor scan (MS1) was performed in the mass range from *m/z* 375 to 1,500 at a resolution of 120,000 with an automatic gain control (AGC) target of 4 × 10^5^ . The peptides were fragmented using HCD with collision energy of 30%, and the DDA-MS2 data were collected at a resolution of 15,000 with an AGC target of 5 × 10^4^.

Raw data were analyzed using Proteome Discoverer ver. 3.1 software (Thermo Fisher Scientific) on the MASCOT Server ver. 2.8 with the following parameters: Cys alkylation: iodoacetamide, Digestion: Trypsin, Species: Homo sapiens or Mus musculus, and FDR (false discovery rate): strict 1% and relaxed 5%. The EMARS products were subjected to MS analysis two times to ensure reproducibility, and potential HCAF candidates were filtered based on the following criteria: (i) molecules directly labeled with biotinyl tyramide; (ii) molecules scarcely detected in the negative control (EMARS products derived from GFP-expressing influenza PR8; abundance ratio of 100); and (iii) molecules classified as membrane proteins according to the UniProt database. The selected molecules are summarized in Tables S1 and S5.

### Western blot

After SDS-PAGE gel analysis of the EMARS products, the gels were subjected to western blot analysis to confirm fluorescence labeling, if necessary. After electrophoresis, the gels were blotted onto an Immobilon-P PVDF membrane (Millipore), followed by blocking with 5% skim milk solution. The membrane was incubated with sheep anti-fluorescein antibody (6400-01; Southern Biotech; 0.5 µg/mL 5% skim milk solution) at room temperature for 1 h followed by HRP-conjugated anti-sheep IgG (HAF016; R&D; 1:5,000 5% skim milk solution) at room temperature for 1 h.

To detect HRP expression in stable HRP-expressing HEK293T packaging cells, each cell pellet collected from a 10 cm dish was solubilized with 20 μL reducing SDS sample buffer. Each 5 μL sample solution was subjected to 10% SDS-PAGE followed by blotting and blocking. The membrane was incubated with goat anti-HRP antibody (123-005-0211; Jackson ImmunoResearch; 1 µg/mL 5% skim milk solution) followed by HRP-conjugated anti-goat IgG (sc-2020; Santa Cruz; 1:5,000 5% skim milk solution) at room temperature for 1 h.

To detect HRP expression in pseudoviruses, each pseudovirus supernatant was enriched using a Lenti-X Concentrator (Takara Bio) as described above. The enriched virus pellet was solubilized in 50 μL reducing SDS sample buffer. Each 15 μL (VSV-G virus) or 2 μL (influenza virus) sample solution was subjected to 8% or 10% SDS-PAGE followed by blotting and blocking. The membrane was incubated with goat anti-HRP antibody (123-005-0211; Jackson ImmunoResearch; 1 µg/mL 5% skim milk solution) followed by HRP-conjugated anti-goat IgG (sc-2020; Santa Cruz; 1:5,000 5% skim milk solution) at room temperature for 1 h.

To detect HA expression in influenza pseudoviruses, each pseudovirus supernatant was enriched using a Lenti-X Concentrator (Takara Bio) as described above. The enriched virus pellet was solubilized in 50 μL reducing SDS sample buffer. Each 2 μL sample solution was subjected to 8% or 10% SDS-PAGE followed by blotting and blocking. The membrane was incubated with anti-influenza HA antibody mixture consisting of avian influenza A virus H5N3 HA (Hemagglutinin) antibody (GTX127299; GeneTex; 2 µg/mL 5% skim milk solution), influenza A virus H3N2 HA antibody (GTX53724; GeneTex; 2 µg/mL 5% skim milk solution), and influenza A virus H1N1 HA antibody (GTX127357; GeneTex; 0.46 µg/mL 5% skim milk solution). The incubations were performed at 4°C overnight. After primary antibody treatment, HRP-conjugated anti-rabbit IgG (W4011; Promega; 1:5,000 5% skim milk solution) was subsequently treated at room temperature for 1 h.

Each membrane was developed with Immobilon Western Chemiluminescent HRP Substrate (Millipore) and analyzed using a ChemiDoc MP Image Analyzer (Bio-Rad). As loading controls, the PVDF membrane after exposure was stained with Coomassie brilliant blue solution, if necessary.

### Transmission electron microscopy (TEM)

To investigate whether HRP expression alters viral morphology or whether HRP is detectable in the virus, each SFM cultured supernatant (ASF104; Ajinomoto) containing Inf-HRP and Inf-GFP pseudoviruses was mixed 1:1 with 4% paraformaldehyde (09154-85; Nacalai Tesque) at room temperature for at least 15 min. Supernatants from untransfected HEK293T cells cultured in SFM were used as negative controls. When labeling HRP on viral particles with gold colloid, prior to fixation, gold colloid (12 nm)-conjugated anti-HRP antibody (LS-C74279; LSBio) was added at a dilution of 1:200 and incubated at room temperature for 30 min. Subsequently, the solution was fixed with 4% paraformaldehyde as described above. A 10 μL aliquot of the fixed sample solution was placed onto a hydrophilic formvar-coated grid (Nisshin EM) and allowed to stand for 10 min, resulting in adsorbing sample molecules onto the membrane surface. The grid was then washed by immersion in a 2% uranyl acetate solution, and the remaining solution was removed with filter paper. The grid was further washed two times in the 2% uranyl acetate solution for 1 s each. Excess stain on the grid was absorbed using a filter paper, and the grid was allowed to air-dry. The prepared samples were examined at 80 kV using a JEM-1400 transmission electron microscope (JEOL).

### Virus attachment assay

We used NRP1, which was identified by proteome analysis, and GPC3, a molecule not listed in the proteome results but previously used in SARS studies (23), as a negative control molecule for the assay. The NRP1 (HG29858-UT; Sino Biological) gene was purchased as inserts into the pCMV3 vector. For GPC3, we utilized pcDNA-GPC3, which was constructed in previous SARS studies (23). To generate HRP-expressing influenza virus (PR8) for the assay, we seeded HEK293T cells in one well of a six-well dish (TPP) and CHO-K1 cells in the remaining five wells. After 48–72 h, the HEK293T cells were transfected with a vector set for producing pseudovirus expressing HRP and the HA of the aforementioned A/Puerto Rico/8/1934 (H1N1: PR8) strain, while the CHO-K1 cells were transfected with expression vectors for NRP1 and GPC3 (200 ng/well) using TransIT-2020 reagent (2 µL/well). After 72 h, 20 µL influenza pseudovirus-containing culture medium was added to each well of CHO-K1 cells, and the cells were incubated at room temperature for 10 min to allow virus attachment. The cells were washed three times with PBS, followed by reaction with 0.05 mM BT (SML2135; Sigma-Aldrich) and 0.0075% H_2_O_2_ in PBS at room temperature for 10 min. After three additional washes with PBS, the cells were collected into plastic tubes and treated with anti-NRP1 antibody (AF3870; R&D Systems; 1.25 µg/mL 0.5% BSA-PBS) and anti-GPC3 antibody (10088-T38; Sino Biological; 1:200 0.5% BSA-PBS), respectively, at room temperature for 20 min. Subsequently, the cells were treated with a mixture of fluorescently labeled secondary antibodies, including Alexa Fluor 647-conjugated anti-sheep antibody (ab150179; Abcam; 5 µg/mL 0.5% BSA-PBS), anti-rabbit antibody (ab150079; Abcam; 5 µg/mL 0.5% BSA-PBS), and anti-mouse antibody (A-21235; Thermo Fisher Scientific; 5 µg/mL 0.5% BSA-PBS), and streptavidin-Alexa Fluor 488 (S32354; Thermo Fisher Scientific; 5 µg/mL 0.5% BSA-PBS) at room temperature for 20 min. After washing the cells with PBS, the fluorescence intensities of Alexa Fluor 488 and Alexa Fluor 647 on the cell surface were analyzed by flow cytometry (FACS) without compensation operation as described above. We compared the fluorescence intensities of Alexa Fluor 488 (which reflects the number of biotin moieties, corresponding to the amount of HRP bound to the cells and thus the amount of virus bound to the cells) and Alexa Fluor 647 (which reflects the expression levels of each candidate HCAF) to determine whether they were correlated. The FlowJo software (ver. 10.10.0; BD Biosciences) was used to digitize the fluorescence of each cell type.

### Data processing

Statistical analyses were performed using Microsoft Excel (version 2505; Microsoft Corporation) and the Perplexity AI software (https://www.perplexity.ai/). The data derived from the virus diameter, virus production, and infection assays were analyzed using a two-sample Student’s *t*-test, with the assumption of equal variance between groups, in Microsoft Excel. The correlation coefficients and *P* values for each sample were calculated using the Pearson correlation coefficient with the assistance of Perplexity AI. Statistical significance tests for the correlation coefficients among the five samples were performed using the Mann–Whitney *U*-test in Perplexity AI. Statistical significance was defined as *P* < 0.05.

## Data Availability

Raw proteomic data and search files for protein identification of HCAFs were deposited in the ProteomeXchange Consortium (ID PXD075131) via the Japan ProteOme STandard (jPOST) Repository/Database (https://jpostdb.org/) (ID JPST004445).

## SUPPLEMENTAL MATERIAL

Supplemental figures jvi.00507-26-s0001.pdf Fig. S1 to S5.

Table S1 jvi.00507-26-s0002.xlsx Raw data of MS analysis using Inf-GFP, Inf-HRP, and VSVG-HRP viruses; first experiment.

Table S2 jvi.00507-26-s0003.xlsx Proteins labeled with Biotin-tyramide; first experiment.

Table S3 jvi.00507-26-s0004.xlsx Raw data of MS analysis using Inf-GFP, Inf-HRP, and VSVG-HRP viruses; second experiment.

Table S4 jvi.00507-26-s0005.xlsx Proteins labeled with Biotin-tyramide; second experiment.

Table S5 jvi.00507-26-s0006.xlsx Proteins reproducibly labeled with Biotin-tyramide in duplicate experiments.

ASM does not own the copyrights to Supplemental Material that may be linked to, or accessed through, an article. The authors have granted ASM a non-exclusive, world-wide license to publish the Supplemental Material files. Please contact the corresponding author directly for reuse.
