# Supplementary material for: Pseudovirus-mediated proximity labeling identifies candidate host cell membrane proteins involved in viral attachment
Source: J Virol. 2026 Apr 29;100(5):e00507-26. doi: 10.1128/jvi.00507-26 (PMC13185619; doi:10.1128/jvi.00507-26)
Supplement: Supplemental figures — Fig. S1 to S5. [file jvi.00507-26-s0001.pdf]

Fig. S1 Generation of HRP-expressing pseudovirus using stable HRP-expressing HEK293T cells

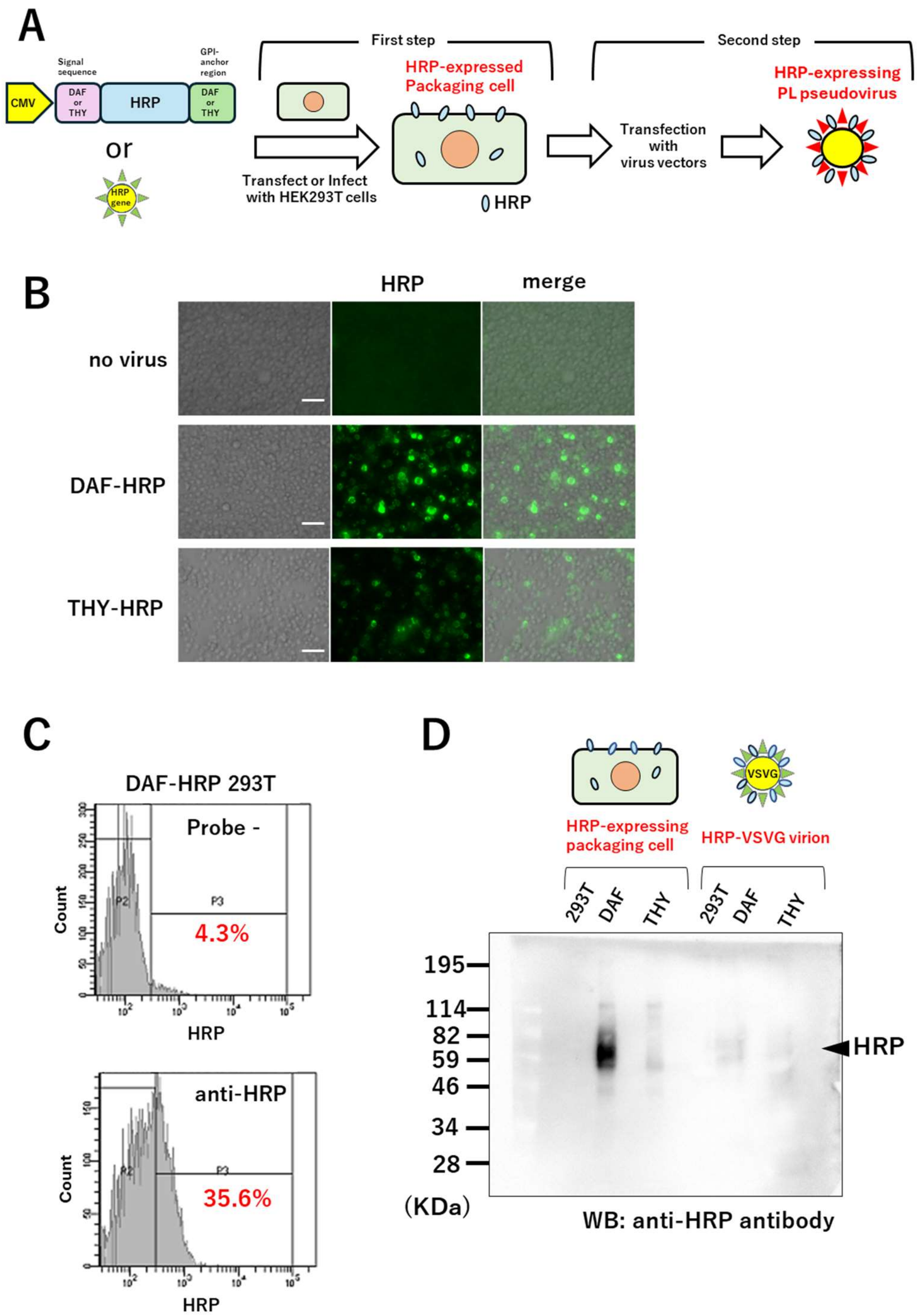

(*A*) Schematic illustration of the procedure for generating pseudoviruses (Two-step method). For the production of HRP-expressing pseudoviruses in Two-step method, stable HRP-expressing HEK293T cells were first established by lentiviruses carrying DAF-HRP or THY-HJRP genes (First step). Using these stable packaging cells, the virus vectors carrying lentivirus essential gene and the spike protein expression vector were transfected for generating PL pseudovirus (Second step). (*B*) Detection of HRP expressions in lentivirus-treated HEK293T cells after 48 hr culture. Following treatment of lentiviruses carrying DAF-HRP or THY-HJRP genes with HEK293T cells, the cells were stained with an anti-HRP antibody followed by Alexa Fluor 488-conjugated second antibody for observation by fluorescence microscopy. Two independent experiments were performed. As a negative control, the cells without pseudovirus treatment were prepared (No virus). The white bar indicates 100 μm. (*C*) Representative FACS analysis for DAF-HRP expression. DAF-HRP-expressing HEK293T cells were treated with a rhodamine-conjugated anti-HRP antibody for staining. The stained cells (anti-HRP) and unstained cells served as negative control cells (Probe -) were both analyzed using FACS. The histogram data was divided into two regions, and gating was applied to calculate the percentage of cells in the high fluorescence intensity area (P3). Two independent experiments were performed. (*D*) Confirmation of HRP expression in both HRP-expressing packaging cells and PL pseudoviruses. Both DAF-HRP (*DAF*) and THY-HRP-expressing packaging cells (*THY*) were subjected to western blot analysis with anti-HRP antibody (left area). HEK293T mock cells were used as negative control cells (*293T*). DAF-HRP (*DAF*) and THY-HRP (*THY*) pseudovirus carrying VSV-G were concentrated and subjected to western blot analysis (right area). The pseudoviruses produced by typical HEK293T packaging cells were used as negative control pseudoviruses (*293T*). The membranes were stained using anti-HRP antibody as described "*Materials and Methods*". Two independent experiments were performed.

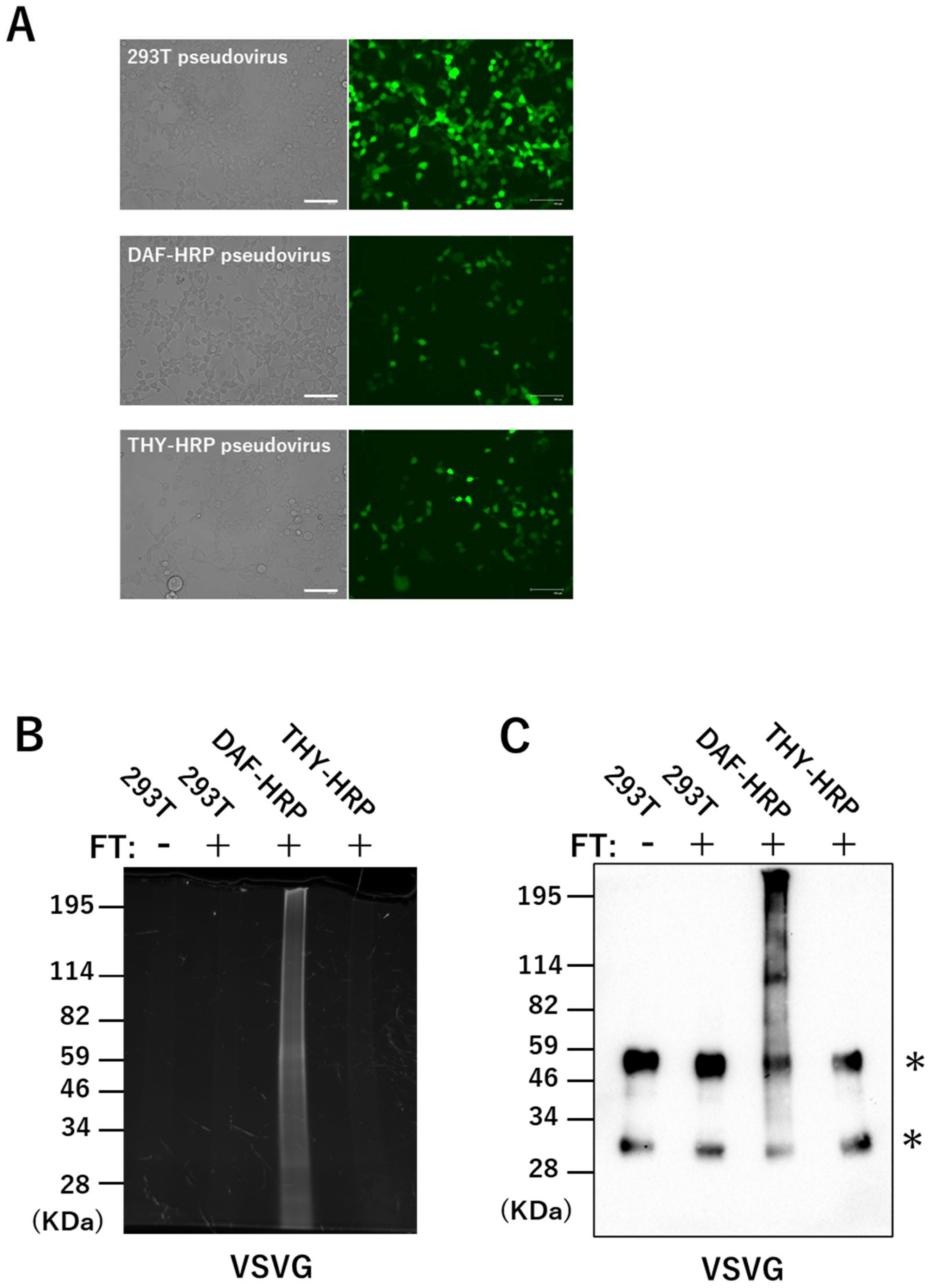

Fig. S2 EMARS reaction using HRP-expressing pseudovirus

(*A*) Immunocytochemistry of pseudovirus-infected HEK293T cells. The VSV-G pseudoviruses carrying GFP gene derived from HEK293T cells, DAF-HRP-expressing HEK293T cells, and THY-HRP-expressing HEK293T cells were respectively infected with HEK293T cells. After 48 hr culture, each treated cell was observed by fluorescein microscopy for GFP expression. Two independent experiments were performed. The white bar indicates 100 μm. (*B, C*) SDS-PAGE and western blot analysis of EMARS products. HEK293T cells were treated with three types of VSV-G pseudoviruses in (*A*) and then performed EMARS reaction. The EMARS products were enriched with anti-fluorescein antibody-Sepharose and then analyzed by SDS-PAGE as described "*Materials and Methods*" (*B*). The gel was subsequently applied to western blot analysis with anti-fluorescein antibody (*C*). Two independent experiments were performed. Asterisks indicate the bands of H and L chain from anti-fluorescein antibody-Sepharose.

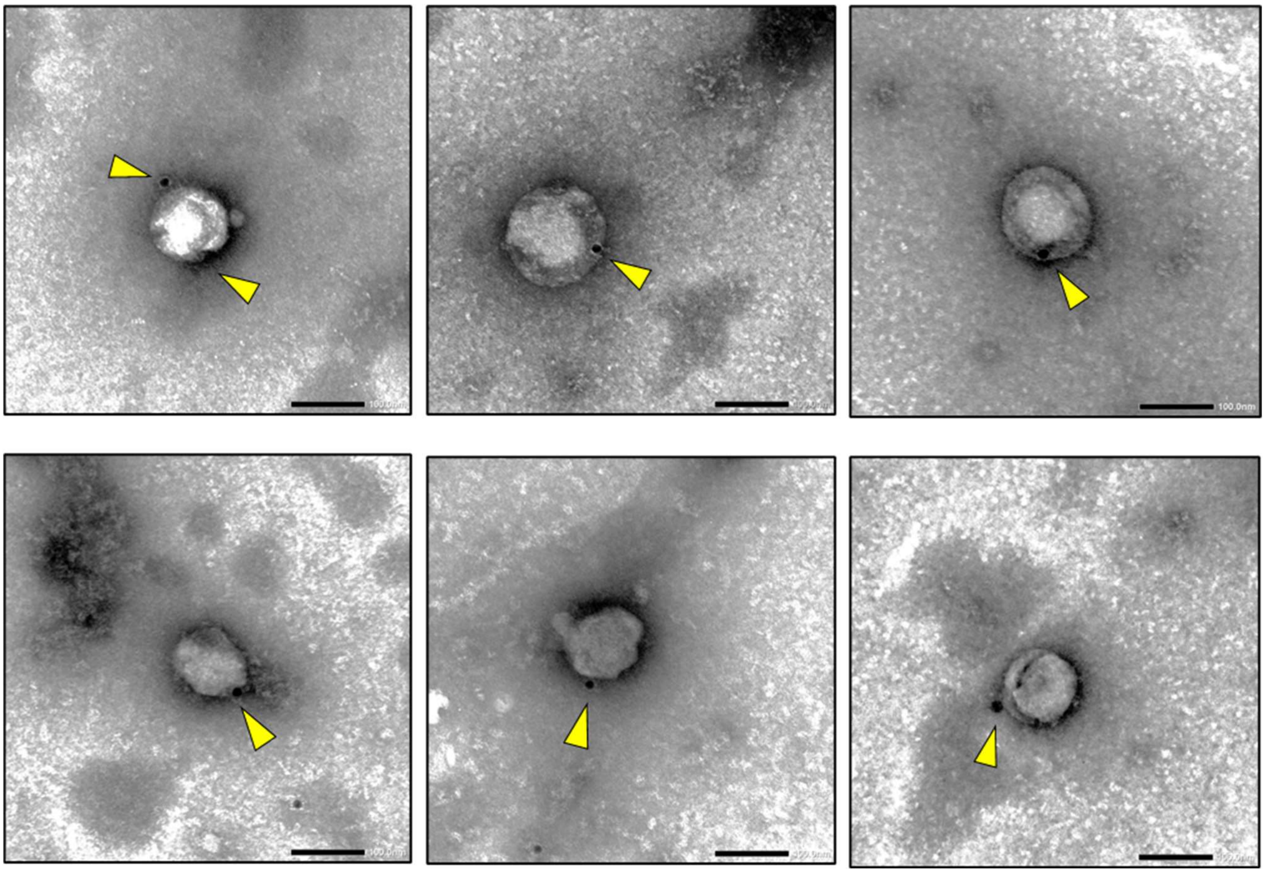

Fig. S3 Morphological observation of pseudoviruses using transmission electron microscopy (TEM)
Inf-HRP virus was treated with anti-HRP gold colloid (12 nm: yellow arrowheads) antibody and then observed by TEM. In addition to Fig. 1E, six of the gold colloid-labeled particles are shown. Scale bar, 100 nm.

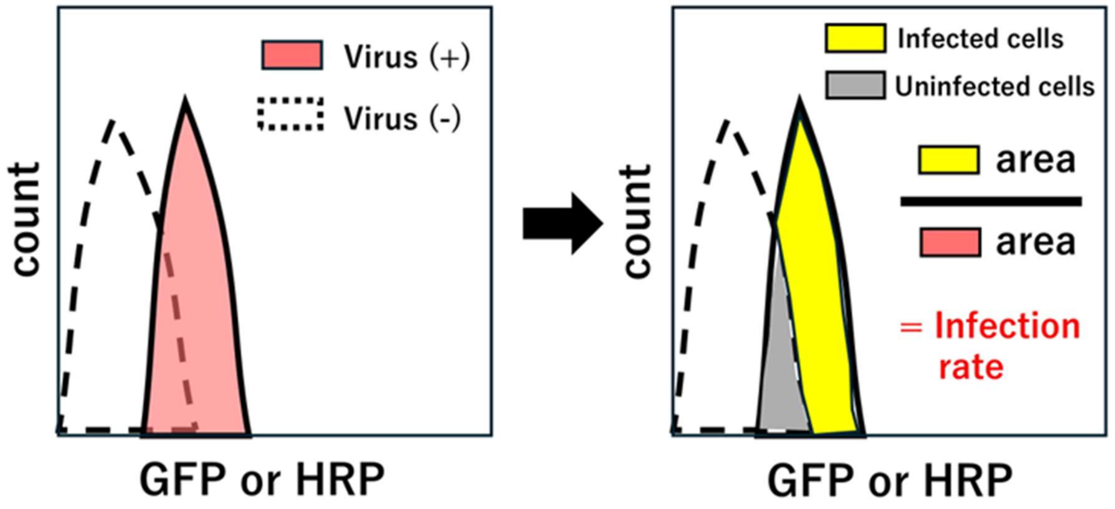

Fig. S4 Calculation of infection rate using FACS analysis data.
Histograms were imported into Fiji (NIH ImageJ), and the areas corresponding to infected and uninfected cells were quantified. The infection rate was calculated based on these area measurements.

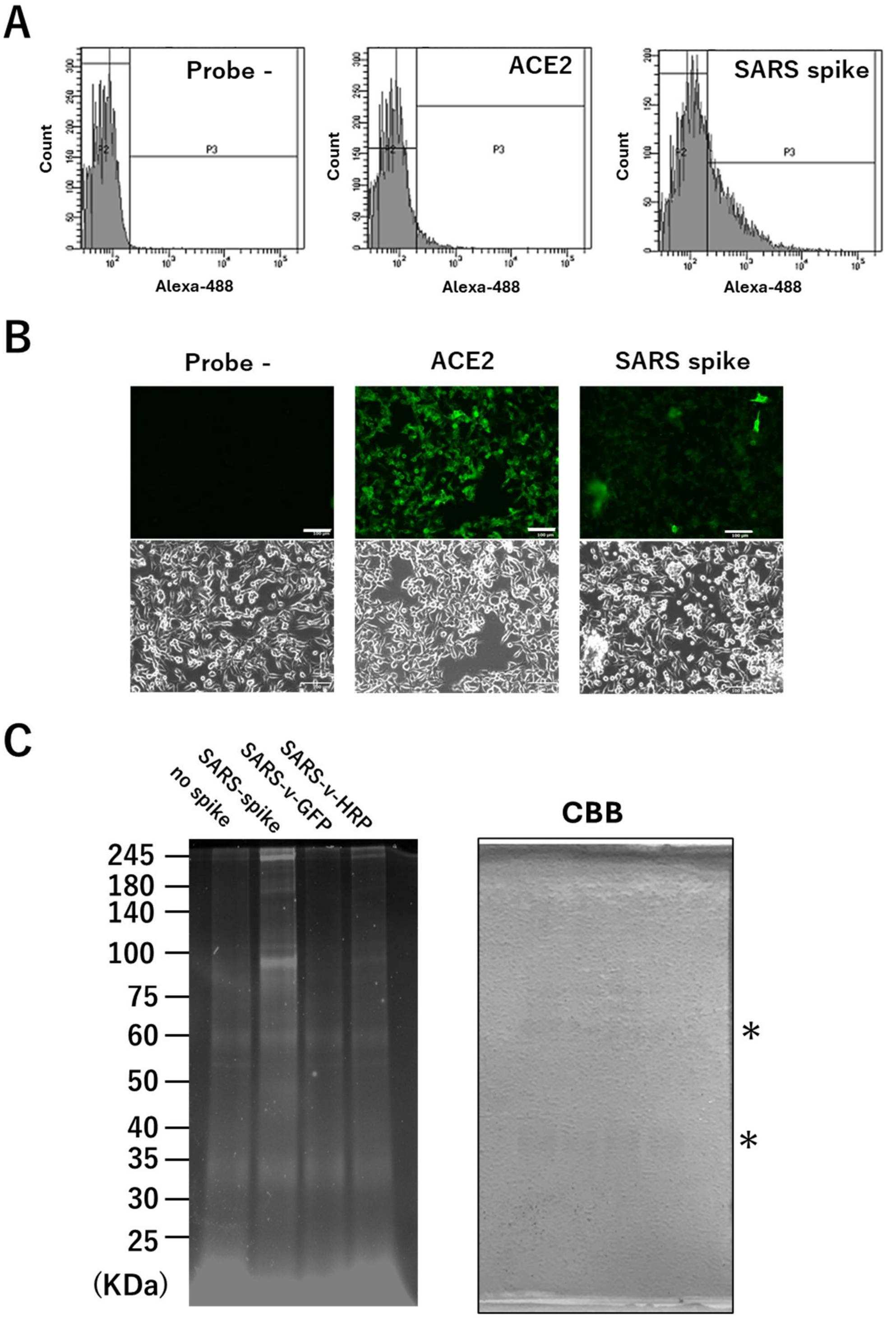

Fig. S5 Differences between EMARS using recombinant spike protein or PL pseudovirus. (*A, B*) Characterization of HeLa-A-T cells as host cells. HeLa-A-T cells were assessed for ACE2 expression (*ACE2*) and spike protein binding (*SARS spike*) using FACS (*A*) and immunocytochemistry (*B*). Cells without antibody and spike protein treatment were served as negative controls (*Probe–*). The white bar indicates 100 µm. (*C*) SDS-PAGE analysis of EMARS products derived from both spike and pseudovirus. HEK293T cells were treated with recombinant spike protein (*SARS-spike*), HRP-expressing pseudovirus carrying SARS-CoV-2 spike proteins (*SARS-v-HRP*), or GFP pseudovirus carrying SARS-CoV-2 spike proteins (*SARS-v-GFP*) as negative control viruses. After thorough washing, FT reagent was added to initiate the EMARS reaction. The EMARS products were enriched with anti-fluorescein antibody-Sepharose and then analyzed by SDS-PAGE as described "*Materials and Methods*". Two independent experiments were performed. CBB staining was used as a loading control; however, due to the purification of EMARS products, no protein bands detectable by CBB were observed except for nearly equal amounts of heavy and light chains (asterisks; derived from antibodies bound to the resin during the enrichment process).
